# Inhibition of BMK1 pathway suppresses cancer stem cells through BNIP3 and BNIP3L

**DOI:** 10.18632/oncotarget.5337

**Published:** 2015-09-29

**Authors:** Chengli Song, Qiang Xu, Kui Jiang, Guangyu Zhou, Xuebin Yu, Lina Wang, Yuting Zhu, Liping Fang, Zhe Yu, Jiing-Dwan Lee, Shi-Cang Yu, Qingkai Yang

**Affiliations:** ^1^ Department of Oncology, The Second Affiliated Hospital of DaLian Medical University, Institute of Cancer Stem Cell, DaLian Medical University, Dalian, Liaoning 116044, China; ^2^ Department of Immunology and Microbial Science, The Scripps Research Institute, La Jolla, CA 92037, USA; ^3^ Institute of Pathology and Southwest Cancer Center, Southwest Hospital, Third Military Medical University, Chongqing 400038, China

**Keywords:** cancer stem cell, BMK1, kinase

## Abstract

Cancer stem cells (CSCs) possess many characteristics associated with stem cells and are believed to drive tumor initiation. Although targeting of CSCs offers great promise for the new generation of therapeutics, lack of the effective drugable target and appropriate pharmacological reagents significantly impedes the development of chemotherapies. Here, we show that the phosphorylation of BMK1 was significantly correlated with not only embryonic and induced pluripotent stem (iPS) cells, but also the CSCs. It was showed that activation of BMK1 by the expression of MEK5D enhanced the self-renew (sphere formation), proliferation (clone formation) and tumorigenic capacity of CSCs. While BMK1 inhibitor, XMD8-92, suppressed these capacities. RNA-seq and microarray analysis revealed that inhibition of BMK1 significantly enhanced the expression of BNIP3 and BNIP3L, which play important roles in cell death. Further study indicated that shRNA-mediated knock down of BNIP3 and BNIP3L impairs the BMK1 inhibitor, XMD8-92-induced suppression of sphere formation and clone formation of CSC. Collectively, these results not only indicate that BMK1 plays an important role in maintaining “stemness” of CSCs, but also implicate that BMK1 might be a potential drug target for CSCs.

## INTRODUCTION

Cancer stem cells (CSCs), as a small fraction of cancer cell population, have been supposed to play key roles in tumor initiation, treatment resistance and recurrence [1, 2]. Therefore, development of specific therapies targeting CSCs is promising for improvement of cancer therapeutics. However, conventional cancer therapeutics tend to preferentially eliminate the non-CSCs, leaving behind residues of CSCs, which can subsequently result in recurrence. Hence, the identification of key CSC pathways and development of appropriate inhibitor are critical for CSC-targeted therapy.

It is well known that the mitogen-activated protein (MAP) kinase signaling pathways play a central role in the decision of cell fate. In mammalian, four MAPK pathways have been discovered and are known as ERK1/2, BMK1 (also called MAPK7 or ERK5), p38 and JNKs pathways [3]. Generally, ERK1/2 and BMK1 are activated by oncogenic signals and involved in promoting cell proliferation and survival; while JNK and p38 are supposed to regulate cell death and proliferation [3, 4]. Although BMK1 is the most closely related to ERK1/2 [5], it seems that ERK5 and ERK1/2 pathways play opposing regulatory roles in stem and progenitor cells [6]. At the meantime, our previous study has showed that deletion of BMK1 results in embryonic lethal, which also suggested that BMK1 might play an important role in maintaining “stemness” [7]. Moreover, deregulated BMK1 signaling has so far been correlated with general properties of human malignancies, including tumorigenesis, chemoresistance [8], proliferation [9] and metastatic potential [10], which are also the capacities of CSCs.

Here, we demonstrated that BMK1 was activated in not only embryonic and induced pluripotent stem (iPS) cells, but also the tumor sphere-enriched CSCs. It was also showed that the phosphorylation of BMK1 by the expression of MEK5D enhanced the sphere formation, clone formation and tumor-initiating capacity (TIC) of CSCs, while the inhibition of BMK1 can abolish these capacities.

## RESULTS

### Phospho-BMK1 was correlated with iPS, embryonic and cancer stem cells

**Figure 1:**
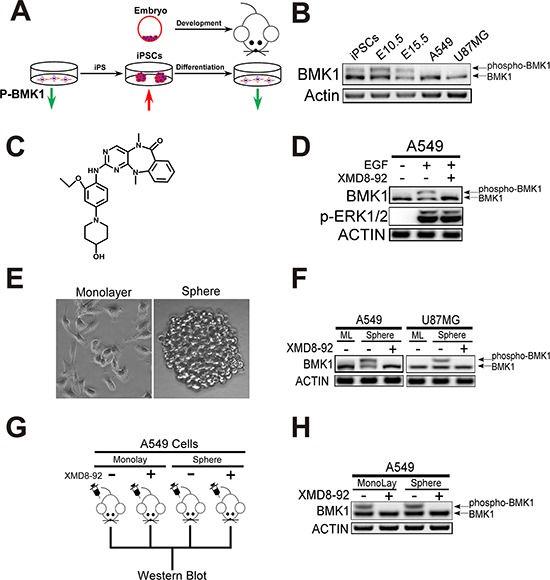
Phospho-BMK1 was correlated with iPS, embryonic and cancer stem cells **A.** Scheme for phospho-BMK1 enhancement in iPS and embryonic cells. **B.** Phospho-BMK1 was associated with the stem cells. Proteins from A549, U87MG, iPS, E10.5 (whole embryo) and E15.5 (whole embryo) cell lysates were resolved by SDS–polyacrylamide gel electrophoresis and phosphorylated BMK1 was detected by mobility retardation [36]. **C.** Chemical structure of BMK1 inhibitor XMD8-92 as described in our previous study [12]. **D.** BMK1 inhibitor, XMD8-92 specifically blocked the activation of BMK1. A549 cells were serum starved overnight followed by treatment with/without 2 μmol/L XMD8-92 for 1 hr. Then these cells were treated with EGF for 15 mins. Phospho-ERK1/2 (T202/Y204), BMK1 and ACTIN were detected by the antibody as noted. **E.** U87MG cells grew in monolayer and sphere as noted. **F.** The phosphorylation of BMK1 was significantly elevated in tumor spheres. U87MG and A549 monolayer/sphere cells were treated with 2 μmol/L XMD8-92 for 1 hr. BMK1 and ACTIN were detected by the antibody as noted. ML: monolayer. **G.** Experimental scheme for A549 xenograft model. Xenograft models were carried out as previously described [12]. Briefly, 1 × 10^4^ cultured A549 monolayer and sphere cells were suspended in DMEM and injected subcutaneously into the right flank of 6-week-old Nod/Scid mice. These tumor-bearing mice were randomized into groups. Mice were injected with/without XMD8-92 (50 mg/kg) for 3 hrs as noted. **H.** The phospho-BMK1 was significantly increased *in vivo* tumor. Proteins from (G) A549 tumor cell lysates were resolved by SDS–polyacrylamide gel electrophoresis and phosphorylated BMK1 was detected by mobility retardation.

Considering that CSCs share a lot of characteristics associated with normal stem cells, it followed logically to investigate the oncogenic pathway, which also plays critical role in stem cells. As described above, previous studies have suggested that BMK1 might play a critical role in maintaining “stemness” of stem and progenitor cells (Figure 1A). To test this, the phospho-BMK1 was evaluated in iPS and whole embryonic (E10.5 and 15.5) cells. As expected, it was found that phospho-BMK1 was significantly elevated in iPS and embryonic stem cells compared with A549 and U87MG cells, most of which are supposed to be non-CSCs (Figure 1B). In our previous study, it has been described that a small-molecule inhibitor of BMK1, XMD8-92 (Figure 1C), can inhibit BMK1 and impair the tumorigenesis (Figure 1D). Using this BMK1 inhibitor, it was found that inhibition of BMK1 impaired the self-renew of iPS cells (Supplementary Figure S1A) and resulted in embryo lethal (Supplementary Figure S1B). These data indicated that BMK1 might increase “stemness”. Based on these, the phosphorylation of BMK1 was evaluated in tumor spheres, which were supposed to enrich the CSCs or even taken as the CSCs [11]. It was found that the phosphorylation of BMK1 was significantly enhanced in tumor sphere cells compared with monolayer cells (Figure 1E), while BMK1 inhibitor XMD8-92 blocked this phosphorylation of BMK1 as expected (Figure 1F). To confirm the correlation of phospho-BMK1 with tumorigenicity, an A549 xenograft model was built to evaluate the phosphorylation of BMK1 as previously described [12]. Briefly, monolayer and sphere A549 cells were injected subcutaneously into mice and treated with/without XMD8-92, respectively (Figure 1G). The resultant tumors from both monolayer and sphere A549 cells showed upregulation of phospho-BMK1 (Figure 1H), which reinforced the important role of BMK1 in tumorigenicity. Collectively, these data indicated that phospho-BMK1 was significantly correlated with the stemness and tumorigenicity of CSCs.

### Inhibition of BMK1 effectively suppressed the self-renew and proliferation of cancer stem cells

**Figure 2:**
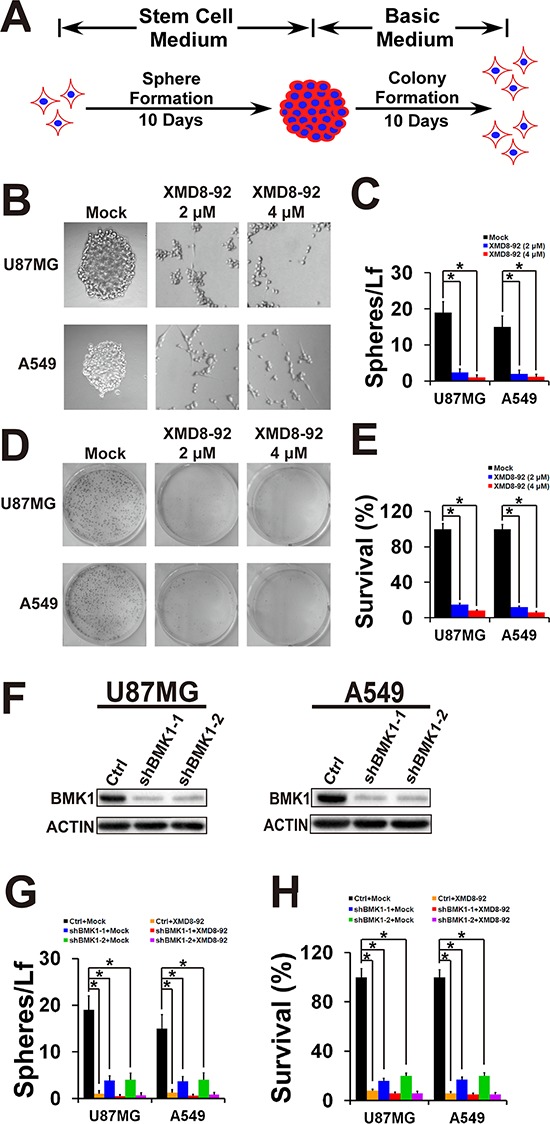
Inhibition of BMK1 effectively suppressed the self-renew and proliferation of cancer stem cells **A.** Scheme for sphere and colony formation assay. Briefly, tumor spheres were cultured in stem cell medium containing DMEM/F12, B27 (1X), EGF (20 ng/ml) and bFGF (20 ng/ml) as previously described [13]. After 10 days, 1 × 10^3^ sphere cells were plated in 6 well dish in DMEM (basic medium), which contained 10% FBS, 2 mM glutamine, 100 U/ml penicillin and streptomycin. **B.** Sphere formation of U87MG and A549 cells treated with vehicle, 2 μmol/L or 4 μmol/L XMD8-92 as noted. **C.** The number of tumor spheres derived from (B) was counted 10 days after seeding Light microscopy × 100. *n* = 5, ± SEM, *p value < 0.01. Spheres/Lf: number of tumor spheres in Light microscopy field. **D.** and **E.** Colony formation of A549 and U87MG spheres. Sphere cells were plated in 6 well dish in DMEM (basic medium) containing 10% FBS. After 10 days, cells were stained with MTT. *n* = 5, ± SEM, *p value < 0.01. **F.** shRNA-mediated knock down of BMK1 in A549 and U87MG cells. BMK1 and ACTIN were detected by the antibody as noted. Sequences of shBMK1–1 and shBMK1–2 were described in Supplementary Table S4. **G.** Sphere formation of the resultant cell lines from (F) as noted. *n* = 5, ± SEM, *p value < 0.01. **H.** Colony formation of the resultant cell lines from (F) as noted. *n* = 5, ± SEM, *p value < 0.01.

To investigate the role of BMK1 in CSCs, sphere and colony formation was carried out to evaluate the self-renew and proliferation of CSCs, respectively (Figure 2A) [11, 13]. For sphere formation assay, tumor cells were cultured in stem cell medium containing DMEM/F12, B27, EGF and bFGF as previously described [13]. After 10 days, sphere cells were plated in basic medium (DMEM contained 10% FBS). As shown in Figure 2B and Figure 2C, XMD8-92 treatment significantly inhibited the sphere formation of U87MG and A549 cells. Similarly, XMD8-92 treatment also significantly impaired the colony formation of U87MG and A549 cells as shown in Figure 2D and Figure 2E. To confirm this, BMK1 was also knocked down in both A549 and U87MG cells using two shRNAs (Figure 2F). The resultant control and shBMK1 cells were treated with/without XMD8-92 as noted. Compared with the control cells, shBMK1 U87MG and A549 cells show reduction of sphere formation (Figure 2G) and colony formation (Figure 2H), which also argued that inhibition of BMK1 effectively suppressed both self-renew and proliferation of cancer stem cells.

### Phosphorylation of BMK1 promoted the proliferation, selfrenewal, and tumorigenicity of cancer stem cells

**Figure 3:**
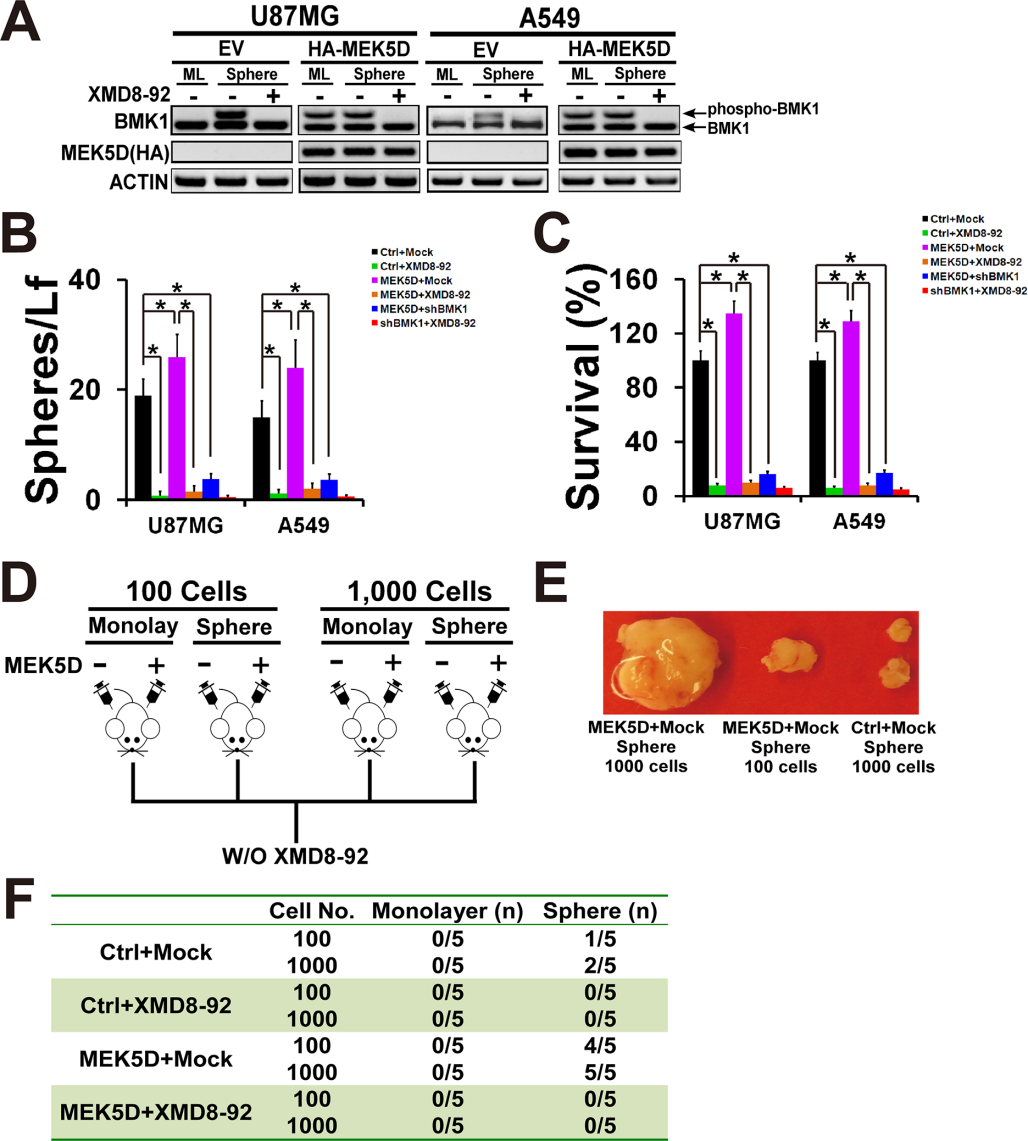
Phosphorylation of BMK1 promoted cancer stem cells **A.** To build the stable expression cell lines, A549 and U87MG cells were transfected with a constitutively active mutant of MEK5 (HA-MEK5D) and vector followed by selection with puromycin. The lysates of stable vector and MEK5D cells were analyzed by western blot using anti-BMK1 and anti-ACTIN antibodies as noted. **B.** Sphere formation of the resultant cell lines as described above. *n* = 5, ± SEM, *p value < 0.01. **C.** Colony formation of the resultant cell lines as described above. *n* = 5, ± SEM, *p value < 0.01. **D.** Experimental scheme for A549 MEK5D xenograft model. Xenograft models were built as previously described [12]. Control and MEK5D A549 cells cultured as monolayer or sphere were suspended in DMEM and injected subcutaneously into the right flank of 6-week-old Nod/Scid mice as noted. And these tumor-bearing mice were randomized into groups and treated with/without XMD8-92 (50 mg/kg) IP twice a day as indicated. **E.** Representative tumors as noted. **F.** Tumorigenecity of MEK5D A549 cells compared with control (vector) A549 cells as noted. *n* = 5.

To further confirm the role of BMK1 in CSCs, a constitutively active mutant of MEK5, MEK5D, was used to phosphorylate BMK1 (Figure 3A) as described in our previous study [4]. As showed in Figure 3A, stable expression of MEK5D enhanced the phosphorylation of BMK1 in U87MG and A549 cells. The resultant stable MEK5D-expressed U87MG and A549 cell lines were used for sphere and colony formation assay with/without XMD8-92 treatment. As expected, expression of MEK5D promoted both sphere and colony formation, which were notablely inhibited by XMD8-92 or shBMK1 (shBMK1-1) (Figure 3B and 3C). Furthermore, an A549 xenograft model was built to evaluate the role of BMK1 in tumorigenicity as described in Figure 3D [12]. Briefly, different amount of monolayer and sphere cells from Ctrl (vector) and MEK5D A549 lines were injected subcutaneously into mice. These tumor-bearing mice were randomized into groups and treated with/without XMD8-92 twice a day as noted. Consistent with the *in vitro* data, analysis of Ctrl and MEK5D A549 xenografts with/without XMD8-92 treatment showed that MEK5D significantly promoted the tumorigenicity, which was impaired by XMD8-92 (Figure 3E and 3F) or shBMK1 knockdown (Supplementary Figure S1D and S1E). Hence, these data indicated that phosphorylation of BMK1 promotes the proliferation, selfrenewal, and tumorigenicity of cancer stem cells.

### Inhibition of BMK1 pathway suppressed the stemness of cancer stem cells through BNIP3 and BNIP3L

**Figure 4:**
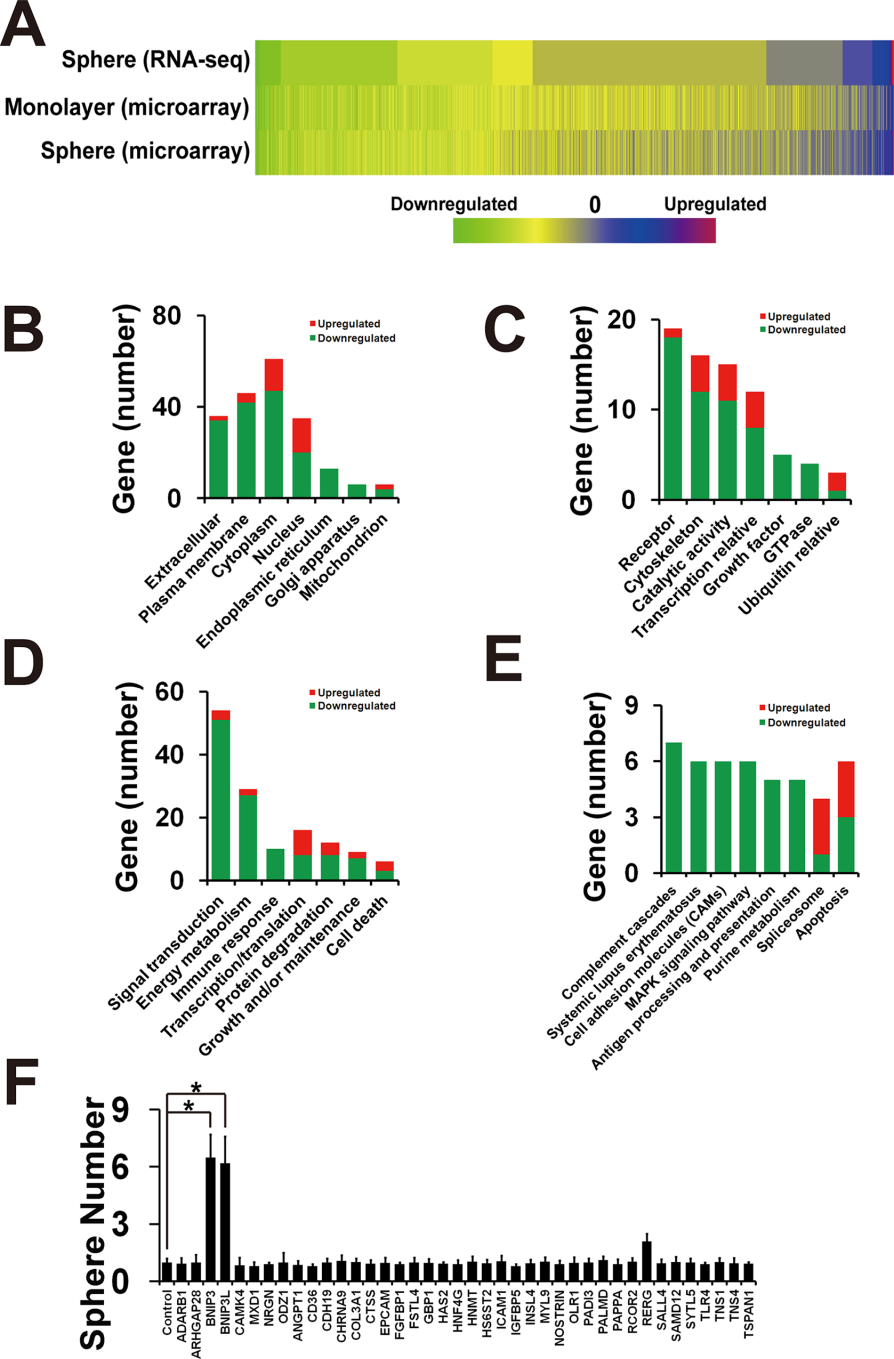
Inhibition of BMK1 pathway suppressed cancer stem cells through BNIP3 and BNIP3L **A.** Heatmap of RNA-seq and microarray assay data from three individual experiments. A549 spheres or monolayer cells were treated with/without 4 μmol/L XMD8-92 for 24 hrs. RNA was extracted using the miRNeasy Mini Kit from QIAGEN (QIAGEN, Hilden, Germany) according to the manufacturer's instruction. The resultant RNA-seq and microarray data are described in Supplementary Table S1 (sphere RNA-seq), Supplementary Table S2 (monolayer microarray) and Supplementary Table S3 (sphere microarray). **B.** Gene number of subcellular localization as noted. **C.** Gene number of molecular function. **D.** Gene number of biological process. **E.** Gene number of pathway analysis by DAVID Bioinformatics Resources 6.7 using default setting. **F.** Sphere formation of 40 shRNA knockdown A549 cells treated with 4 μmol/L XMD8-92 as noted. pLKO.1-scramble shRNA was taken as control and normalized as 1. *n* = 3, ±SEM, *p value < 0.01.

To uncover the mechanisms of BMK1-mediated enhancement of CSCs, we used both RNA-seq and microarray to identify the genes whose expression was changed after XMD8-29 treatment (Figure 4A). A549 sphere cells treated with/without XMD8-92 were analyzed by RNA-seq, while both monolayer and sphere cells treated with/without XMD8-92 were analyzed by microarray. Further studies of subcellular localization (Figure 4B), molecular function (Figure 4C), biological process (Figure 4D) and pathway analysis (Figure 4E) indicated that inhibition of BMK1 led to enhance the expression of cell death-associated genes (including BNIP3 and BNIP3L). Furthermore, 40 genes, which showed the significant alteration in both RNA-seq and microarray, were knocked down in A549 cells using shRNAi. Then the resultant shRNA A549 cell lines were used for the XMD8-92-induced suppression assay (Figure 4F). It was found that shBNIP3 and shBNIP3L significantly blocked XMD8-92-induced suppression of sphere formation (Figure 4F).

**Figure 5:**
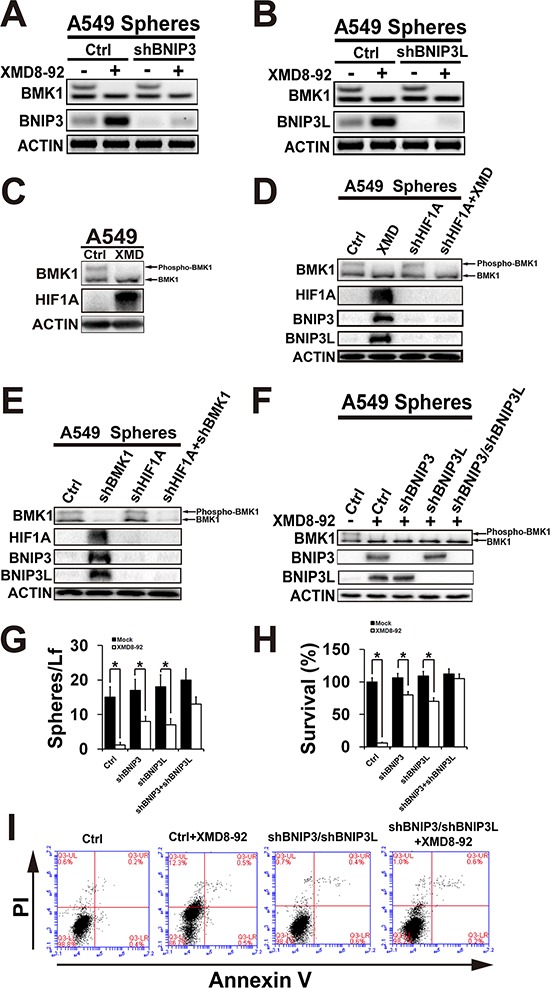
Inhibition of BMK1 pathway suppressed cancer stem cells through BNIP3 and BNIP3L **A.** and **B.** Inhibition of BMK1 enhanced BNIP3 and BNIP3L. Control, shBNIP3 or shBNIP3L A549 sphere cells were treated with/without 4 μmol/L XMD8-92 for 24 hrs as noted. The resultant lysates of sphere cells were analyzed by western blot using anti-BNIP3, anti-BNIP3L and anti-ACTIN antibodies as noted. **C.** A549 sphere cells were treated with/without 4 μmol/L XMD8-92 for 4 hrs as noted. The resultant lysates of sphere cells were analyzed by western blot using anti-BMK1, anti-HIF1A and anti-ACTIN antibodies as noted. **D.** Control and shHIF1A A549 sphere cells were treated with/without 4 μmol/L XMD8-92 for 24 hrs as noted. The resultant lysates of sphere cells were analyzed by western blot using appropriate antibodies as noted. **E.** The lysates of control, shHIF1A, shBMK1 and shHIF1A/shBMK1 A549 sphere cells were analyzed by western blot using appropriate antibodies as noted. **F.** Control, shBNIP3, shBNIP3L and shBNIP3/shBNIP3L A549 sphere cells were treated with/without 4 μmol/L XMD8-92 for 24 hrs as noted. The resultant lysates of sphere cells were analyzed by western blot using appropriate antibodies as noted. **G.** Sphere formation of control, shBNIP3, shBNIP3L and shBNIP3/shBNIP3L A549 cell lines. *n* = 5, ± SEM, *p value < 0.01. **H.** Colony formation of control, shBNIP3, shBNIP3L and shBNIP3/shBNIP3L A549 cell lines. *n* = 5, ± SEM, *p value < 0.01. **I.** Control, and shBNIP3/shBNIP3L A549 sphere cells were treated with/without XMD8-92 for 36 hrs as noted. The resultant cells were analyzed by flow cytometry using PI and Annexin V staining as noted.

As expected, further study indicated that BMK1 inhibitor XMD8-92 significantly enhanced BNIP3 (Figure 5A) and BNIP3L (Figure 5B) in A549 sphere cells. Considering both BNIP3 and BNIP3L were the downstream of Hypoxia-inducible factor 1α (HIF1α) [14], the role of BMK1 in the regulation of HIF1α was investigated in A549 sphere cells. Treatment of A549 sphere cells with BMK1 inhibitor for 4 hrs significantly increased HIF1α, which suggested that inhibition of BMK1 was able to stabilize HIF1α (Figure 5C). Further study indicated that shRNA knockdown of HIF1α significantly impaired the upregulation of BNIP3 and BNIP3L, which was induced by BMK1 inhibitor (Figure 5D) or shRNA knockdown (Figure 5E). At the meantime, double knockdown of BNIP3 and BNIP3L (Figure 5F) notablely blocked XMD8-92-induced suppression of sphere and colony formation of CSCs (Figure 5G and 5H). Most importantly, it was also found that double knockdown of BNIP3 and BNIP3L significantly impaired XMD8-92-induced apoptosis of A549 CSCs (Figure 5I). Taken together, these experiments provide the evidence of feasibility for BMK1 inhibition in the setting of cancer stem cells through BNIP3 and BNIP3L.

## DISCUSSION

Despite of their small quantity, CSCs are proposed to play a crucial role in the initiation, progression and recurrence of cancer. Development of specific therapies targeted at CSCs holds hope for improvement of survival of cancer patients. Therefore, elucidation of the pathways that regulate the maintenance and survival of CSCs is important for the development of novel therapies.

In many kinds of cancer, it has be reported that deregulated BMK1 signaling is correlated with general capacities of CSCs, including tumorigenesis, chemoresistance [8], proliferation [9] and metastatic potential [10]. Traditional deletion of BMK1 results in embryonic lethal, which reinforces the potential important role of BMK1 in stem cells. At the mean time, conditional knockout of BMK1 in various tissues (such as neurons, hepatocytes, cardiomyocytes, and T and B cells) has no obvious effect on the development, behavior, reproduction and aging of the mouse [7], suggesting that BMK1 might be a potential drug target for CSC-targeted therapy.

In this study, it was described that the phospho-BMK1 was significantly enhanced in not only embryonic and iPS cells as expected, but also the CSCs. It was showed that activation of BMK1 enhanced the sphere formation (self-renew), clone formation (proliferation) and tumor-initiating capacity (TIC) of CSCs, while inhibition of BMK1 impaired these capacities. Moreover, it was found that inhibition of BMK1 significantly enhanced BNIP3 and BNIP3L, which play critical roles in cell death. And this enhancement of BNIP3 and BNIP3L is through HIF1α. Further study showed that knock down of BNIP3 and BNIP3L hampered the XMD8-92-induced suppression of sphere formation and clone formation of CSC. Therefore, inhibition of BMK1 pathway suppressed cancer stem cells through BNIP3 and BNIP3L.

BNIP3 and BNIP3L are related to the BH3-only family and promote both cell death and autophagy [14]. Consistent with their ability to induce cell death, BNIP3 and BNIP3L inhibit tumor growth especially in hypoxia conditions. Previous studies have indicated that hypermethylation of the BNIP3 promoter was found in pancreatic cancer [18], and the BNIP3L gene was infrequently mutated in a panel of primary breast and ovarian tumors [19]. Since BNIP3 and BNIP3L promote cell death, it is not surprising that upregulation of BNIP3 and BNIP3L were reported to block cancer stem cells in multi types of cancer [20]. However, an intriguing aspect of the biology of BNIP3 and BNIP3L is their role in autophagy, apart from the role in cell death. Despite the controversy, which still exists regarding the characteristics of CSCs, a correlation between autophagy and CSCs has been suggested. Autophagy has been shown to be involved in the maintenance of CSCs in various tumors, such as breast cancer [21] and pancreatic cancer [22]. Therefore, BNIP3 and BNIP3L exhibit a dual nature—they promote both cell death and autophagy, which has a protective effect in some settings. This dual nature effects were also observed in HIF1, the well known upstream of BNIP3 and BNIP3L.

As one of the most famous and well studied transcription factors, HIF1 plays a central role in the cellular response to hypoxia. HIF1 regulates the transcription of a broad range of genes that facilitate responses to the hypoxic environment, including genes regulating angiogenesis, erythropoiesis, cell cycle, metabolism and cell death [23]. The HIF1 heterodimeric complex consists of two subunits, HIF1A and HIF1B. Under normoxic conditions, two critical prolines in HIF1A (Pro564 and Pro402) can be hydroxylated by proline hydroxylase (PHD). Hydroxylation of HIF-1A leads to a conformational change that promotes binding to VLH (von Hippel Lindau protein) and ubiquitin mediated degradation [23]. Although many studies have indicated that moderate HIF1A and hypoxia has been heavily implicated in stem cells and CSCs [24, 25], HIF1A and hypoxia are also well known to promote cell death through at least two mechanisms [26]. First, some HIF1A targets are genes capable of inducing cell death, including RPT801 [27], BNIP3 [14, 28] and BNIP3L [14] as described above. Second, HIF1A-induced cell death may be stemmed from the cooperation between the hypoxia pathway and p53 pathway [29, 30], both of which are significantly regulated by BMK1 [17, 31]. In fact, many studies have indicated that HIF1A functions as a tumor suppressor instead of a tumor promoter in many cancers [29, 32]. Hence, this double-edged sword of HIF1A should be carefully manipulated in cancer therapies. Interestingly, although inhibition of BMK1 enhanced HIF1A as described above, knockout of BMK1 impairs angiogenesis and results in vascular collapse [7]. We hypothesize that this might due to the cooperation between BMK1\P53 pathway [17, 31] and BNIP3 (and/or BNIP3L) pathway. But more work is still needed.

Collectively, this study strengthened the notion that BMK1 plays an important role in CSCs, and blocking the BMK1 pathway may be an effective approach for targeting CSCs.

## MATERIALS AND METHODS

### Cell culture, transfection and induction of pluripotent stem cells

The A549 and U87MG cells were purchased from the ATCC and maintained in Dulbecco's modified Eagle's medium (DMEM) (basic medium), which contained 10% heat-inactivated FBS, 2 mM glutamine, 100 U/ml penicillin and streptomycin at 37°C under a humidified atmosphere of 5% CO_2_. Transfections were performed using Lipofectmin 2000 (Gaithersburg, MD, USA) according to the manufacturer's instruction.

The induction of pluripotent stem cells was performed as described in the previous study [33]. Briefly, Oct4-EGFP MEF cells were infected by four retroviruses containing Oct4, Sox2, Klf4 and c-Myc. After three days, the resultant MEF cells were plated on the MEF feeder cells and maintained in mouse embryonic stem cell medium. GFP-positive clones were taken as the iPS cells.

### Tumor sphere culture

Tumor spheres were cultured and characterized as previously described [13, 34]. U87MG and A549 cells were seeded in 24-well plates at 1 × 10^4^ cells/well and maintained in Dulbecco's modified Eagle's medium (DMEM) (basic medium), which contained 10% heat-inactivated FBS, 2 mM glutamine, 100 U/ml penicillin and streptomycin at 37°C under a humidified atmosphere of 5% CO_2_ for 18 hrs. Thereafter, cells were cultured in stem cell medium containing DMEM/F12 (Gibco, USA), B27 (1x, Gibco), recombinant human epidermal growth factor (rhEGF, 20 ng/ml; Sigma, USA) and basic fibroblast growth factor (bFGF, 20 ng/ml; Upstate, USA).

### Colony formation assay

For colony formation assays, 1 × 10^3^ cells dissociated from tumor spheres were plated in 6 well dish in Dulbecco's modified Eagle's medium (DMEM), which contained 10% heat-inactivated FBS, 2 mM glutamine, 100 U/ml penicillin and streptomycin at 37°C under a humidified atmosphere of 5% CO_2_. After 10 days, the cells were incubated in 0.5 ml MTT (3-(4,5)-dimethylthiahiazo (−z-y1)-3,5-di-phenytetrazoliumromide) solution (final concentration: 0.5 mg/ml) for 3 hrs. Then the medium was replaced by 0.2 ml DMSO. The plates were agitated for 15 min and the optical density of the solution in the wells was measured at 490 nm using EnSpire Multimode Plate Reader (PerkinElmer Inc, USA).

### Reagents

Anti-HA (Cat. number: 3724), anti-BMK1 (Cat. number: 3372), anti-phosho-ERK1/2 (Thr202/Tyr204) (Cat. number: 4376), anti-BNIP3 (Cat. number: 13795), anti-BNIP3L (Cat. number: 12396), anti-HIF1A (Cat. number: 14179), anti-Annexin V (Cat. number: 8555) and anti-ACTIN (Cat. number: 4967) antibodies were from Cell Signaling (Beverly, MA, USA). HA-tagged MEK5D-expressing vectors and BMK1 inhibitor (XMD8-92) were described in our previous study [12]. Sequence of shRNA (MISSION^®^ shRNA Library, sigma, USA) used in this study was described in Supplementary Table S4. pLKO.1-scramble shRNA was used as shRNAi control.

### Immunoblotting

Immunoblotting was carried out as described in our previous study [12, 35]. Briefly, cells were washed with cold PBS for 3 times and harvested in RIPA buffer (1X PBS, 1% NP40, 0.5% sodium deoxycholate, 0.1% SDS, phosphatase inhibitor cocktail (Roche, Indianapolis, IN), 0.1 mg/ml PMSF and 1 mM sodium orthovanadate). Proteins from cell or tissue lysates were resolved by 8%, 10% or 12% SDS-polyacrylamide gel electrophoresis, transferred to nitrocellulose membrane, blocked in 5% nonfat milk and blotted with the appropriate antibody.

### Cell apoptosis assay

Control and shBNIP3/shBNIP3L A549 sphere cells were treated with/without 4 μmol/L XMD8-92 for 36 hrs, and collected by trypsinization and centrifugation at 300 G for 5 mins. The resultant cells were washed twice with cold PBS and resuspended in 100 μL Annexin-binding buffer. Then Annexin V (final concentration: 1 μg/ml) and PI (final concentration: 30 μg/ml) were added into 100 μL cell suspension. Then cells were incubated at room temperature for 15 minutes. The fluorescence intensity was detected with the flow cytometry.

### Xenograft models

The following animal-handling procedures were approved by the Animal Care and Use Committee of DaLian Medical University. Xenograft models were built as described in the previous study [12]. Briefly, cells as indicated were injected subcutaneously into the right/left flank of 6-week-old NOD/SCID mice. These tumor-bearing mice were randomized into groups and treated W/O XMD8-92 (50 mg/kg) IP twice a day as indicated. Tumor size was measured by caliper and tumor volume was calculated by using the formula: 0.52 × *L* × *W^2^*, where *L* was the longest diameter and *W* was the shortest diameter.

### Statistical analysis

*p* values were calculated using the Student's *t*-test.

## SUPPLEMENTARY FIGURE AND TABLES








