# Supplementary material for: Inhibition of BMK1 pathway suppresses cancer stem cells through BNIP3 and BNIP3L
Source: Oncotarget. 2015 Sep 29;6(32):33279–89. doi: 10.18632/oncotarget.5337 (PMC4741765; doi:10.18632/oncotarget.5337)
Supplement: Supplementary file 1 [file oncotarget-06-33279-s001.pdf]

SUPPLEMENTARY FIGURE AND TABLES

A

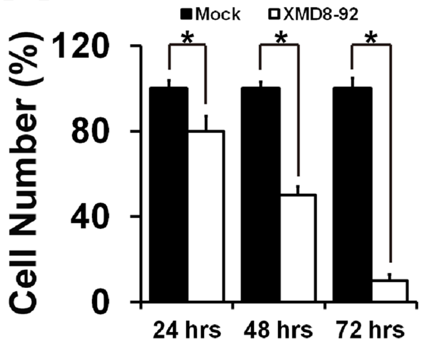

B

	Embryonic Lethal at (n)
E10.5	E12.5 (4/5)<br>E13.5 (1/5)
E15.5	E17.5 (2/5)<br>E18.5 (3/5)

C

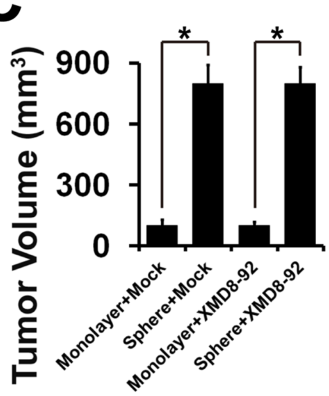

D

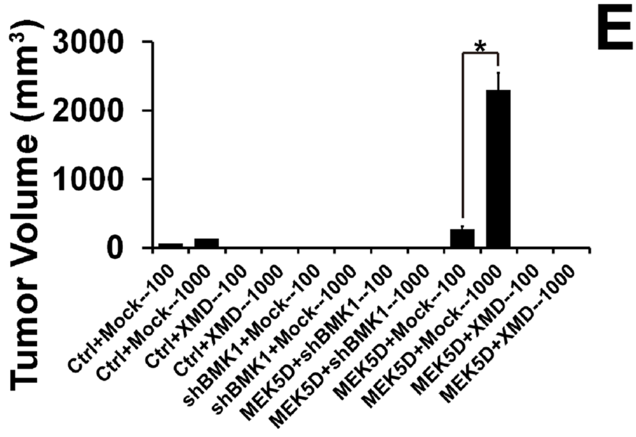

E

	Cell No.	Monolayer (n)	Sphere (n)
Ctrl+Mock	100	0/5	1/5
	1000	0/5	2/5
Ctrl+XMD8-92	100	0/5	0/5
	1000	0/5	0/5
shBMK1+Mock	100	0/5	0/5
	1000	0/5	0/5
MEK5D+shBMK1	100	0/5	0/5
	1000	0/5	0/5
MEK5D+Mock	100	0/5	4/5
	1000	0/5	5/5
MEK5D+XMD8-92	100	0/5	0/5
	1000	0/5	0/5

Supplementary Figure S1: A. iPS cells were treated with/without 4 μmol/L XMD8-92 as noted. Then the cell number was evaluated with MTT assay. The number of iPS cell without treatment was taken as 100%. **B.** Pregnant mice (E10.5 and 15.5) were treated with 50 mg/kg XMD8-92 IP twice a day. The resultant embryonic lethal was showed as noted. $n = 5$. **C.** 1×10^4 cultured A549 monolayer and sphere cells were suspended in DMEM and injected subcutaneously into the right flank of 6-week-old Nod/Scid mice. And these tumor-bearing mice were randomized into groups. Mice were injected with/without XMD8-92 (50 mg/kg) for 3 hrs as noted. Tumor size was measured by caliper and tumor volume was calculated by using the formula: $0.52 \times L \times W^2$, where L was the longest diameter and W was the shortest diameter. $n = 5$, ± SEM, *p value < 0.01. **D.** Control, shBMK1 (shBMK1-1) and MEK5D A549 cells cultured as monolayer or sphere were suspended in DMEM and injected subcutaneously into the right flank of 6-week-old Nod/Scid mice as noted. And these tumor-bearing mice were randomized into groups and treated with/without XMD8-92 (50 mg/kg) IP twice a day as indicated. Tumor size was measured as described above. $n = 5$, ± SEM, *p value < 0.01. **E.** Tumorigenecity of control, shBMK1 (shBMK1-1) and MEK5D A549 cells as noted. $n = 5$.

See the Supplementary Table S1: Sequence of shRNA.

See the Supplementary Table S2: RNA-seq of A549 sphere cells.

See the Supplementary Table S3: Microarray of A549 monolayer cells.

Supplementary Table S4: Microarray of A549 sphere cells

Gene	Sequence
ADARB1	CCGGCCCGTGATGATCTTGAACGAACTCGAGTTCGTTCAAGATCATCACGGGTTTTTG
ANGPT1	CCGGTGGAAATCCCTCCGGTGAATACTCGAGTATTCACCGGAGGGATTTCCATTTTTG
ARHGAP28	CCGGGCCATTCAACTCAACAATCAACTCGAGTTGATTGTTGAGTTGAATGGCTTTTTTG
BNIP3	CCGGGCCACGTCACTTGTGTTTATTCTCGAGAATAAACACAAGTGACGTGGCTTTTT
BNIP3L	CCGGCAGTCAGAAGAAGAAGTTGTACTCGAGTACAACTTCTTCTTCTGACTGTTTTT
CAMK4	CCGGAGAAAGTTAAAGGTGCAGATACTCGAGTATCTGCACCTTTAACTTTCTTTTTT
CD36	CCGGACGGCTGCAGGTCAACCTATTCTCGAGAATAGGTTGACCTGCAGCCGTTTTTTTG
CDH19	CCGGCGAACCCAATGGTAGTCTTAACTCGAGTTAAGACTACCATTGGGTTCGTTTTTG
CHRNA9	CCGGGGGTGACTGGCCTCTAGTTTACTCGAGTAAACTAGAGGCCAGTCACCCTTTTTTG
COL3A1	CCGGCCGTTCTCTGCGATGACATAACTCGAGTTATGTCATCGCAGAGAACGGTTTTTG
CTSS	CCGGCACAGTTGCATAAAGATCCTACTCGAGTAGGATCTTTATGCAACTGTGTTTTT
EPCAM	CCGGCGCGTTATCAACTGGATCCAACTCGAGTTGGATCCAGTTGATAACGCGTTTTTG
FGFBP1	CCGGGAGCTCTCTCTGCACATTCTTCTCGAGAAGAATGTGCAGAGAGAGCTCTTTTTTG
FSTL4	CCGGCCTCCGATTTGACGATTACAACTCGAGTTGTAATCGTCAAATCGGAGGTTTTTG
GBP1	CCGGCGGAAATTCTTCCCAAAGAAACTCGAGTTTCTTTGGGAAGAATTTCCGTTTTTG
HAS2	CCGGTGGAATCACAGCTGCTTATATCTCGAGATATAAGCAGCTGTGATTCCATTTTTTG
HNF4G	CCGGACATCAATGATCGGCAGTATGCTCGAGCATACTGCCGATCATTGATGTTTTTTTG
HNMT	CCGGCAATGCTAAGATGCTCATTATCTCGAGATAATGAGCATCTTAGCATTGTTTTTTG
HS6ST2	CCGGGCCTCTAGTGTAGAGATCAATCTCGAGATTGATCTCTACACTAGAGGCTTTTTG
ICAM1	CCGGCGGCTGACGTGTGCAGTAATACTCGAGTATTACTGCACACGTCAGCCGTTTTTG
IGFBP5	CCGGCGACGAGAAAGCCCTCTCCATCTCGAGATGGAGAGGGCTTTCTCGTCGTTTTTG
INSL4	CCGGCATGCCTGAGAAGACATTCACCTCGAGGTGAATGTCTTCTCAGGCATGTTTTTG
MXD1	CCGGGCACCAGCATCAAGAGAATAACTCGAGTTATTCTCTTGATGCTGGTGCTTTTT
MYL9	CCGGCCACATCCAATGTCTTCGCAACTCGAGTTGCGAAGACATTGGATGTGGTTTTTG
NOSTRIN	CCGGCCGACTTATCAAGTCCTAAATCTCGAGATTTAGGACTTGATAAGTCGGTTTTTG
NRGN	CCGGCTCCAAGCCGGACGACGACATCTCGAGATGTCGTCGTCCGGCTTGGAGTTTTTG
ODZ1	CCGGGTGTCAGCCCAAGGCTATAATCTCGAGATTATAGCCTTGGGCTGACACTTTTTTG
OLR1	CCGGGGACAGAGGCCATTCCGAAATCTCGAGATTTCGGAATGGCCTCTGTCCTTTTTG
PADI3	CCGGCACTCTGAAATCATCCATTTGCTCGAGCAAATGGATGATTTCAGAGTGTTTTTTG
PALMD	CCGGCAGATATAATATCGTTCATTCCTCGAGGAATGAACGATATTATATCTGTTTTTTG

Gene	Sequence
PAPPA	CCGGTTGGCAGTGTGTACCAGTATTCTCGAGAATACTGGTACACACTGCCAATTTTTG
RCOR2	CCGGCAAGGAGAAGCATGGCTACAACTCGAGTTGTAGCCATGCTTCTCCTTGTTTTTG
RERG	CCGCATTGTTTCTGGCCTCTAATAGCTCGAGCTATTAGAGGCCAGAAACAATTTTTTG
SALL4	CCGGCCGAACCAACACATCCATTAACTCGAGTTAATGGATGTGTTGGTTCGGTTTTTG
SAMD12	CCGGGAAAGACCACCTTACTATTAGCTCGAGCTAATAGTAAGGTGGTCTTTCTTTTTG
SYTL5	CCGGCGTTTCAAGCAAGTCAATGTTCTCGAGAACATTGACTTGCTTGAAACGTTTTT
TLR4	CCGGGTGGTTCCTAATATTACTTATCTCGAGATAAGTAATATTAGGAACCACTTTTTG
TNS1	CCGGTTGCAATGAAGCGGTTCTATGCTCGAGCATAGAACCGCTTCATTGCAATTTTTG
TNS4	CCGGCAATGACCTCATCCGACACTTCTCGAGAAGTGTCGGATGAGGTCATTGTTTTTG
TSPAN1	CCGGCCAGTCTATTAAACCCTTGATCTCGAGATCAAGGGTTTAATAGACTGGTTTTTG
HIF1A	CCGGGTGATGAAAGAATTACCGAATCTCGAGATTCGGTAATTCTTTCATCACTTTTT
shBMK1-1	CCGGCCCTAATGCTTTCGATGTGGTCTCGAGACCACATCGAAAGCATTAGGGTTTTT
shBMK1-2	CCGGCTTCGATGTGACCTTTGACGTCTCGAGACGTCAAAGGTCACATCGAAGTTTTT
